# Long-Term Oral Administration of *Capsicum baccatum* Extracts Does Not Alter Behavioral, Hematological, and Metabolic Parameters in CF1 Mice

**DOI:** 10.1155/2012/196358

**Published:** 2012-12-17

**Authors:** Aline Rigon Zimmer, Bianca Leonardi, Eduardo Rigon Zimmer, Eduardo Kalinine, Diogo Onofre de Souza, Luis Valmor Portela, Grace Gosmann

**Affiliations:** ^1^Pharmaceutical Sciences Graduate Program, Faculty of Pharmacy, Federal University of Rio Grande do Sul (UFRGS), Ipiranga Avendia 2752, 90610-000 Porto Alegre, RS, Brazil; ^2^Post-Graduate Program in Biological Science, Department of Biochemistry, ICBS, Federal University of Rio Grande do Sul (UFRGS), Ramiro Barcelos Street 2600, 90035-003 Porto Alegre, RS, Brazil

## Abstract

Our group showed that crude ethanol (CE) and butanol (BUT) extracts of *Capsicum baccatum* presented anti-inflammatory and antioxidant properties. Furthermore, the flavonoid and total phenolic contents were positively correlated with both of these properties observed for *C. baccatum* extracts. The present study demonstrated that 60 days of oral administration of CE and BUT (200 mg/kg) in mice did not cause significant differences in the following parameters evaluated: hematological profile, body weight and relative weight of visceral organs, systemic lipid profile, glucose homeostasis (GTT), kidney and hepatic biochemical markers, and spontaneous locomotion and anxiety-like behavior. Altogether, these results indicate for the first time that the long-term oral administration of *C. baccatum* extracts does not affect specific aspects of CF1 mice physiology, suggesting their safety, building up the venue to test their efficacy in animal models underlying persistent activation of oxidative and inflammatory pathways.

## 1. Introduction

*Capsicum* species, from Solanaceae family, are native to the tropical and humid zones of central and south America and include peppers of significant economic value and potential pharmacological application. The fruits vary widely in size, shape, flavor, and sensory heat. The five main species in the genus are *C. annuum*, *C. baccatum, C. chinense*, *C. frutescens, *and *C. pubescens*. These peppers are widely used as spices in the food industry and in a broad variety of medicinal applications worldwide [1, 2].

*Capsicum annuum*, *C. chinense,* and *C. frutescens* are remarkable sources of antioxidant compounds, including capsaicinoids [3, 4] and phenolic compounds, particularly flavonoids [5]. The consumption of these components has potential health benefits due to their activity as free-radical scavengers, which may help prevent inflammatory diseases and pathologies associated with oxidative damage, such as atherosclerosis and Alzheimer's disease [6–8]. These *Capsicum *species exert anti-inflammatory, antioxidant, antiplatelet, antihypertensive, hypoglycemic, and hypocholesterolemic properties *in vitro* and *in vivo *models [8–13]. The main pungent component, capsaicin, has been used clinically for its analgesic and anti-inflammatory properties [14, 15]. 

The red pepper *C. baccatum *var. *pendulum* is widely consumed in food preparations. Reports about the chemical composition of this species are very scarce, especially for this variety. Although the potential pharmacological properties of *C. baccatum *have been also less explored, there are some studies that suggest antioxidant and anti-inflammatory activities of the crude juice [16–18]. A crude juice of *C. baccatum *fruit presented anti-inflammatory effects in a pleurisy model when 2 and 20 g/kg were administered via i.p. and s.c. to rats [19]. In a recent study, we prepared a crude ethanol extract (CE) of *C. baccatum* fruit and, after fractionation, a butanol extract (BUT) enriched in bioactive substances was obtained [20]. We showed that the oral acute administration of 200 mg/kg of BUT extract exhibited the highest antioxidant and anti-inflammatory activities in mice and this extract did not contain capsaicin by HPLC analysis. Furthermore, the flavonoid and total phenolic contents were positively correlated with the antioxidant and anti-inflammatory properties observed for CE and BUT [20]. Although the use of plant extracts as an alternative to conventional medications is widespread, the safety of the extracts needs to be determined before human use [21]. As in this case the constituent concentration intake of these enriched extracts is significantly higher than in the fruit, and considering their potential pharmacological properties previously described, it is relevant to evaluate their long-term oral administration adverse effects. Accordingly, in this study we evaluated the effects of *Capsicum baccatum* extracts on behavioral, hematological, and metabolic parameters of mice.

## 2. Materials and Methods

### 2.1. Plant Material and Extraction

*Capsicum baccatum* var. *pendulum* (Willd.) Eshbaugh (Solanaceae) fruit was obtained from a cultivated area in the city of Turuçu, Rio Grande do Sul, Brazil. This plant was certified by the Germoplasm Bank of the Brazilian Government Research Institute EMBRAPA (Empresa Brasileira de Pesquisa Agropecuária, Pelotas, RS, Brazil) which maintains botanical and chemical uniformity. A voucher specimen (number P278) was identified and deposited at the Herbarium of the Brazilian Government Research Institute EMBRAPA. The fruit of red pepper was dried in a circulating air stove (40°C) and triturated to powder. The fruit was extracted with 70% ethanol (plant : solvent, 1 : 10, w/v) under reflux for 4 hours to obtain the crude ethanol extract (CE). Then the fruit was also submitted to successive extractions in a soxhlet apparatus in order to obtain the dichloromethane, butanol (BUT), and aqueous dried extracts as described previously [20].

### 2.2. Phytochemical Study

The ethanol and butanol extracts were characterized according to the previous studies [20] as follows: the characterization of the phenolic compounds (quercetin and rutin) and capsaicin in *C. baccatum* was performed by HPLC on Agilent instrument (serie 1200) equipped with photodiode array detector (G1322A), autosampler (G1329A), and Agilent ChemStation software. An Ace RP-18 column (250 mm × 4.0 mm i.d., particle size 5 *μ*m) and a linear gradient starting from methanol : acetonitrile : water (15 : 15 : 70) then changed to methanol : acetonitrile : water (30 : 30 : 40) over 25 min, were used. Capsaicin was determined at 280 nm and rutin and quercetin at 254 nm. The total phenolic content was estimated using the Folin-Ciocalteu method customized for 96-well microplates. Gallic acid was used to obtain the calibration curve, and the results were expressed as milligrams of gallic acid equivalents per gram of dried extract (GAE/g). The total content of flavonoids was determined by the AlCl_3_ colorimetric method, using quercetin as standard. Results were expressed as milligrams of quercetin equivalents per gram of dried extract (QE/g).

### 2.3. Animals

Adult 2-month-old CF1 male mice weighing 25–35 g were used. The animals were maintained under controlled temperature (22 ± 2°C) and humidity (55% ± 10%) conditions on a 12 h light-dark cycle (7 : 00 AM and 7 : 00 PM) with free access to standard commercial diet and distilled water. To avoid social isolation two animals were maintained per cage [22]. All experiments complied with the international standards for animal protection and those of the Brazilian College of Animal Experimentation. The Ethical Committee on animal use of the Universidade Federal do Rio Grande do Sul, Brazil, approved all experiments (number 19446).

### 2.4. Experimental Design

The animals were randomized into three groups of 10 animals: distilled water (control group), ethanol extract (CE), and butanol extract (BUT). *C. baccatum* extracts (200 mg/kg body weight) were administered daily by gavage for 60 days. The extracts and dose of 200 mg/kg were selected considering that they presented the higher antioxidant and anti-inflammatory activities in our previous study [20]. Clinical signs of toxicity, general appearance, and mortality were monitored daily during the experimental period. Mean body weight gain and food intake were calculated and compared among groups. Body weights were recorded weekly throughout the study period. Mean daily food consumption was calculated twice per week by subtracting the weight of the remaining food from the weight of the supplied food. All behavioral tasks were performed between 1 : 00 PM and 5 : 00 PM Twenty-four hours after the behavioral tasks, the animals were anesthetized (ketamine:xylazine, 100 : 10 mg/kg, i.p.) to blood sampling for hematological and biochemical analysis. After that, animals were quickly sacrificed, dissected, and subjected to necropsy examination.

### 2.5. Open Field Task

The open field task is a widely used model for the evaluation of spontaneous locomotion and exploratory activities, as well as indicator of anxiety-like behavior [23]. The apparatus was a black-painted box (50 × 50 cm) surrounded by 50 cm high walls. The experiments were conducted in a quiet room under low-intensity light (12 lx). On the 59th day of administration, each mouse was placed in the center of the arena, and the distance travelled (total and central zone), time spent in the central zone, and mean speed were measured for 10 min. To analyze anxiety-like behavior, we created a virtual central zone (30 × 30 cm) using the analysis software and the time in the central zone was used as an indicator of anxiety-like behavior [24]. A video camera positioned above the arena was used to record all experimental sessions. The analysis was performed using a computer-operated tracking system (ANY-maze, Stoelting, Woods Dale, IL). 

### 2.6. Elevated Plus-Maze Task

The elevated plus-maze task measures anxiety-like behavior in rodents and was performed as previously described [25]. The apparatus was made of wood and consisted of four elevated arms (70 cm from the floor × 30 cm long × 5 cm wide) arranged in cross-like disposition and separated by a central zone (5 cm long × 5 cm wide). Two opposite arms were enclosed by 10 cm high walls, with an open roof, and the other two were open (no walls). The experiments were conducted in a sound-attenuated and temperature-controlled room under dim red light illumination. On the 60th day of administration, each mouse was placed individually on the central zone of the plus-maze facing one of the open arms (no walls) and recorded with a video camera for 5 min. The time spent in the open and closed arms, the number of entries into the arms, the total distance travelled, and the mean speed were analyzed. The time spent in open arms was considered a measurement of anxiety-like behavior. The analysis was performed using a computer-operated tracking system (ANY-maze, Stoelting, Woods Dale, IL).

### 2.7. Pathological Examination

All animals were firstly subjected to necropsy by examination of macroscopic external aspect of organs. Then mice were quickly dissected, and the stomach, liver, brain, heart, lung, and kidneys were excised and weighted individually. Fat tissues from the retroperitoneal and epididymal regions were dissected and weighed as previously described [26]. Organ/tissue absolute weight was compared with the final body weight of each mouse on the day of sacrifice to determine the relative organ/tissue weight (absolute organ/tissue weight (g) × 100/animal body weight (g)).

### 2.8. Hematological and Biochemical Analysis

The following hematological parameters were determined by using a semiautomatic blood analyzer (MS4, USA): hemoglobin (Hb), red blood cell (RBC) count, hematocrit (HCT), white blood cell (WBC) count, mean corpuscular volume (MCV), mean corpuscular hemoglobin (MCH), and mean corpuscular hemoglobin concentration (MCHC). For biochemical determinations, these were evaluated: total cholesterol, HDL-cholesterol, LDL-cholesterol, triglycerides, total protein, albumin, alanine aminotransferase (ALT), alkaline phosphatase (ALP), lactate dehydrogenase (LDH), creatinine, and urea. Glucose homeostasis was determined using the glucose tolerance test (GGT), which was performed five days prior to animal sacrifice to avoid metabolic and behavioral alterations. A glucose solution (2 mg/g i.p.) was injected into mice fasted for 12 h, and blood was collected from a small puncture on the tail at 0, 30, 60, and 120 min after injection. Blood glucose level was measured with glucometer (AccuChek Active, Roche Diagnostics, USA), and the area under the curve was used to compare the glucose tolerance among groups [7]. All biochemical determinations were performed using commercial kits (Labtest, MG, Brazil) in a Spectramax M5 (Molecular Devices, USA).

### 2.9. Statistical Analysis

Statistical analysis was performed using a one-way analysis of variance (ANOVA) followed by a Bonferroni test for multiple comparisons. We used the Software GraphPad Prism 5.0 for this analysis. The results are expressed as the mean ± standard error of mean (SEM). Differences were considered significant at *P* < 0.05.

## 3. Results

### 3.1. Phytochemical Study

**Figure 1:**
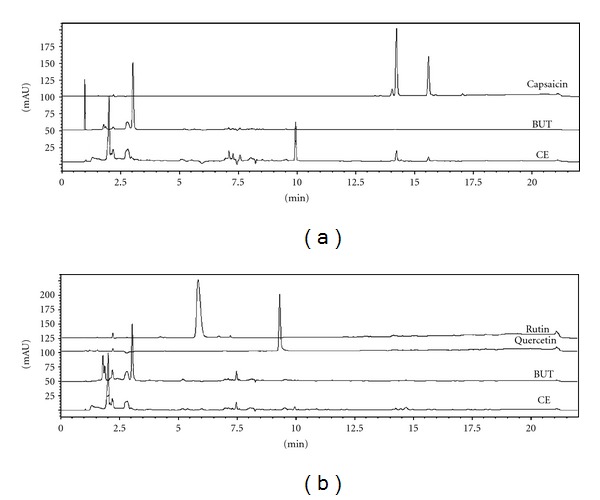
HPLC chromatograms of CE and BUT extracts of* C. baccatum*: (a) detection at 280 nm to capsaicin (Rt = 14.20 min); (b) detection at 254 nm to rutin and quercetin (Rt: 5.80 and 9.26 min, resp.). CE: crude ethanol extract; BUT: butanol extract.

The total phenolic content of *C. baccatum* was the same in CE and in BUT extracts, 180.08 ± 3.76 mg and 187.51 ± 2.34 mg GAE/g, respectively (*P* > 0.05). In contrast, the flavonoid contents present in BUT extract (54.68 ± 2.92 mg of QE/g) were significantly higher compared to CE extract (34.36 ± 4.04 mg QE/g) (*P* < 0.05). HPLC analysis did not identify the presence of flavonoids quercetin and rutin in any samples obtained from *C. baccatum *(Figure 1(b)). Furthermore, capsaicin was not detected in BUT extract (Figure 1(a)) [20].

### 3.2. Open Field Task

**Figure 2:**
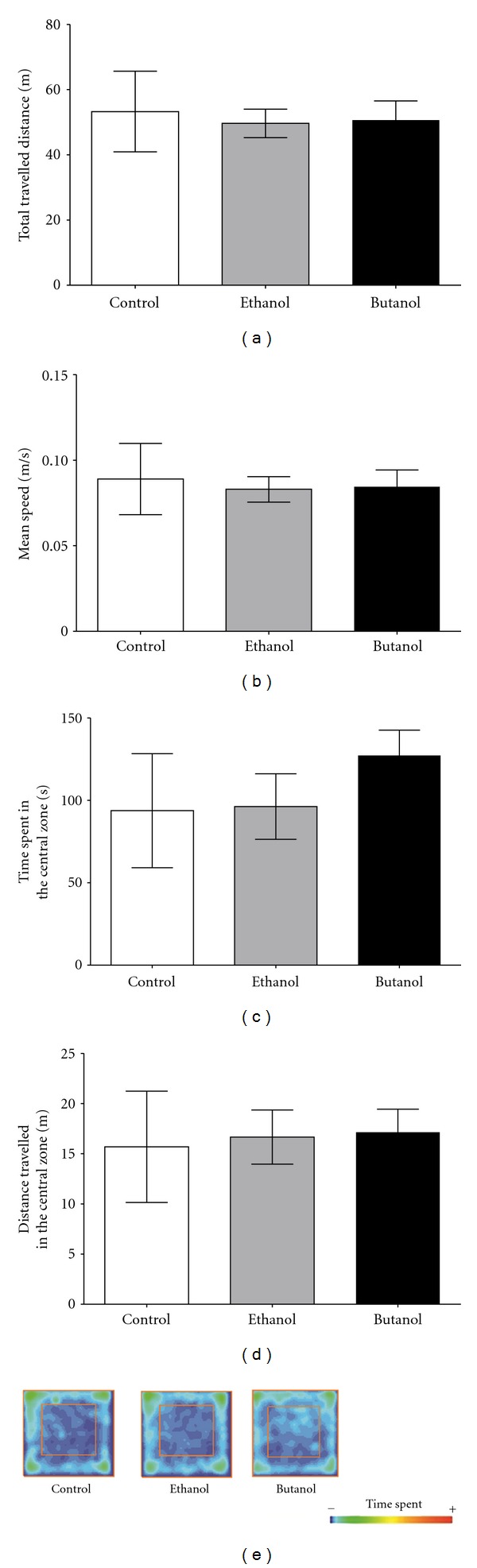
Open field task performed after sixty days of oral administration of *C. baccatum *extracts (200 mg/kg). Total distance travelled (a), mean speed (b), time spent in the central zone (c), and the distance travelled in the central zone of the apparatus (d). Representative occupancy plots obtained by video-tracking software (ANY-mazeH, Stoelting, CO, USA) (e). The results are presented as the mean ± SEM (*n* = 10) using one-way ANOVA. No significant differences were observed among groups.

After the long-term administration of 200 mg/kg of CE and BUT there were no statistical differences among the groups in distance traveled (Figure 2(a)), mean speed (Figure 2(b)), time spent in the central zone (Figure 2(c)), and total distance travelled in central zone (Figure 2(d)). The representative occupancy plots (Figure 2(e)) illustrated a similar exploratory behavior profile among groups during 10 min recording.

### 3.3. Elevated Plus-Maze Task

**Figure 3:**
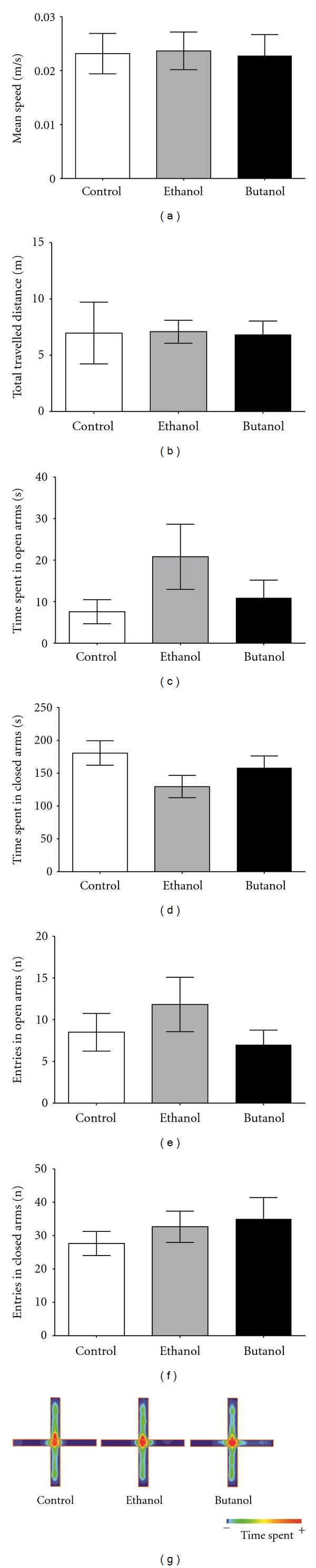
Elevated plus-maze task performed after the administration of *C. baccatum* extracts (200 mg/kg). Mean speed (a), total travelled distance (b), time spent in the open arms (c), time spent in the closed arms (d), number of entries into the closed arms (e), and the number of entries into the open arms (f). Representative occupancy plots obtained by video-tracking software (ANY-mazeH, Stoelting, CO, USA) (g). The results are presented as mean ± SEM (*n* = 10) after analysis by one-way ANOVA. No significant differences were observed among the groups.

There were no differences among groups in mean speed (Figure 3(a)), total distance travelled (Figure 3(b)), time spent in open arms (Figure 3(c)), closed arms (Figure 3(d)), and entries in open arms (Figure 3(e)) and in closed arms (Figure 3(f)). The representative occupancy plots (Figure 3(g)) illustrated a similar occupancy profile of the central zone and open and closed arms of the plus-maze apparatus among groups. These results reinforced the data obtained in the open field task (Figure 2) in which CE and BUT did not cause anxiety-like behavior.

### 3.4. Oral Toxicity Study

**Figure 4:**
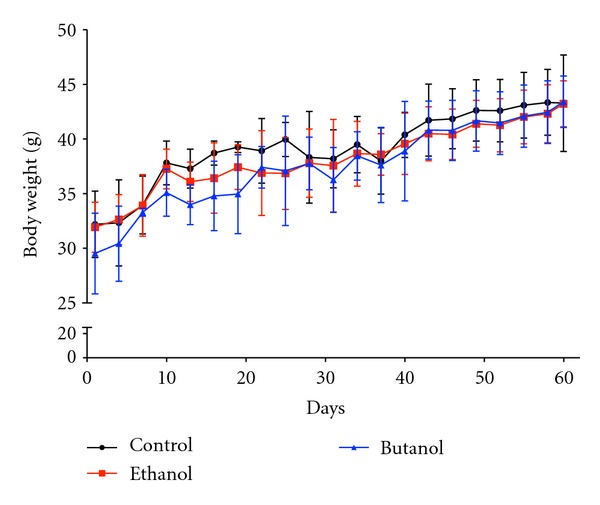
Growth curves of CF1 mice orally treated with *C. baccatum *extracts (200 mg/kg) during sixty days. No significant differences were observed among the groups.

After 60 days of treatment, no clinical signs of toxicity, including hair loss, piloerection, changes in skin, eyes, or oral mucosa, or death, were observed in both treated groups. All animals appeared healthy at the end of the experiment. Visceral examinations of the control and treated mice revealed no visible lesions. There was no accumulation of the extracts, signs of hyperemia, or ulcerations in the gastric mucosa. The body weight increase throughout the administration period was similar in all groups (Figure 4). In addition, there was no statistical difference among groups in final mean body weight and food intake during the administration period. Furthermore, no significant alterations in the relative weights of the kidney, heart, brain, lung, stomach, or epididymal and retroperitoneal fat pads were observed among treated and control groups (Table 1). There was a reduction in the relative liver weight in animals that received CE extract; however at macroscopic examination we did not visualize signs of lesion.

### 3.5. Hematological and Biochemical Parameters

**Figure 5:**
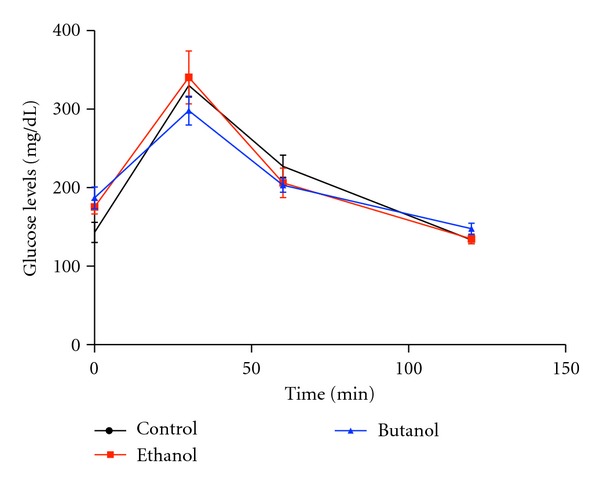
Glucose tolerance test: mice fasted for 8 h received glucose (2 mg/g of body weight, i.p.). Blood samples from the tail vein were taken at 0, 30, 60, and 120 min. Repeated measures of analysis of variance (ANOVA) were used to evaluate the statistical significance. The results are expressed as mean ± SEM No significant difference was observed among groups (*P* > 0.05).

There were no significant differences among groups in hematological parameters analyzed: hemoglobin, red blood cells count, hematocrit, MCV, MCH, MCHC, WBC count, lymphocyte, and neutrophils content (Table 2). Furthermore, there was no significant difference among groups in biochemical parameters analyzed: ALT, ALP, total proteins and albumin, LDH, triglycerides, cholesterol, HDL and LDL, urea, and creatinine (Table 3) at the end of the experiment. Moreover, extracts administration did not cause alterations in glucose tolerance test (Figure 5, *P* > 0.05).

## 4. Discussion

 The red pepper *C. baccatum* is widely used as spice and the few studies on its pharmacological activities were performed using crude extract or juice [16–19]. Previously our group demonstrated that CE and the enriched BUT extract showed the high antioxidant and anti-inflammatory activities. These latter extracts presented higher total phenolic and flavonoid contents than other *Capsicum* species, whose compounds were considered the main responsible for the antioxidant and anti-inflammatory properties imparted to *C. baccatum*. Another relevant issue raised by the former work revealed that BUT extract does not contain capsaicin, one component of other *Capsicum* species associated to anti-inflammatory activity. However, even without detectable presence of capsaicin in BUT, it still maintains significant anti-inflammatory effect. This latter extract represents a new potential therapeutic opportunity to identify novel, more effective, and safety components. Herein, CE and BUT at the daily dose of 200 mg/kg were chosen for investigating their effects on baseline aspects of mice physiology, considering their pharmacological activities described previously [20].

The present study demonstrated that long-term oral administration of CE and BUT had no negative effects on hematologic, metabolic, and behavioral outcomes in normal male CF1 mice. Some features in the normal physiology of rodents resemble to humans and can be used to understand the response to toxic/therapeutic agents, diseases, and pharmacological strategies. Usually mice show a high degree of exploratory behavior necessary to search for food and territory dominance and establish social interactions [27]. In the open field task, CE and BUT administration did not affect total distance traveled, mean speed, time, and distance travelled in the center zone. Similarly, in the elevated plus-maze task, no differences were observed among the groups; they presented similar mean speed, total distance travelled, time spent, and number of entries in open and closed arms. Although in the plus-maze task CE group seems to spend more time in the open arms compared to other groups, the difference did not reach statistical significance. These results indicate that CE and BUT did not cause alterations in spontaneous locomotion and anxiety-like behavior, making it possible for mice to maintain social interactions and reach water and food. In accordance with this, no significant changes in food consumption, body weight gain/final weight, and mortality ratio were observed among groups. Further, during the course of administration, we did not observe mouth lesions or ulcerations caused by gavage procedures or reaction to extracts. Overall, these parameters were considered a preliminary indication that mice had normal absorption of nutrients, hormonal signaling, metabolic and organ-specific functions, which resulted in similar growth and development. In addition, a normal macroscopic appearance was noted in all organs, and there was no difference in organ/body weight among groups.

In this context, we conducted a more precise hematological, metabolic, and tissue assessments, searching for signs of undesirable effects induced by long-term administration of CE and BUT. We confirmed that biochemical profile related to lipid metabolism (serum triglycerides, total cholesterol, HDL, and LDL-cholesterol) and glucose homeostasis (GTT) was similar among groups. There was no indication of cytotoxicity induced by the extracts on hematopoietic red and leucocytes cells. Further, under macroscopic evaluation, there was no sign of alterations in kidney and liver by CE and BUT extracts, which was confirmed by the levels of serum biochemical markers of function/damage of these organs. CE showed decreased liver weight with no significant changes in serum markers of liver damage (ALT, ALP) and functionality (total proteins, albumin, and urea) compared to other groups, which reinforces the lack of structural or functional abnormalities. However, this issue requires caution and additional examinations on liver through histochemical and immunohistochemical investigations.

This study provides evidence that sixty-days administration of *C. baccatum *extracts (CE and BUT) has a pharmacological level of safety. From these data, it is possible to envisage studies to understand its mechanism of action and obtain more data on the reproductive toxicity, genotoxicity, and carcinogenicity in order to proceed to clinical evaluation.

## 5. Conclusion

This work demonstrated for the first time that 60 days of oral administration of crude ethanol and butanol extracts of *C. baccatum* did not affect specific aspects of CF1 mice physiology. In fact, animals showed normal locomotor ability, blood cells counts, lipid and glucose homeostasis, as well as tissue-specific markers of functionality/damage suggesting a level of pharmacological safety. These normal outcomes build up the venue to test the effective dose of these extracts in animal models underlying persistent activation of oxidative and inflammatory pathways for a long-term period. 

## Figures and Tables

**Table 1:** Effects of *C. baccatum* extracts on body weight (g), the relative weight of visceral organs and tissues (g/100 g b.w.), and food consumption (g/day) after a 60-day study in mice (200 mg/kg, *per os*).

Parameters	Control	Ethanol	Butanol
Initial weight (g)	33.7 ± 5.72	33.3 ± 4.03	30.7 ± 3.72
Final weight (g)	44.2 ± 4.68	43.1 ± 2.68	43.4 ± 6.63
Weight gained (g)	10.5 ± 3.63	9.8 ± 2.52	12.7 ± 3.33
Weight gained (%)	31.2	29.4	41.4
Food intake (g)/day	9.5 ± 1.9	9.0 ± 1.7	11.0 ± 2.2
Relative epididymal fat pad weight (%)	1.50 ± 0.48	1.02 ± 0.31	1.18 ± 0.34
Relative retroperitoneal fat pad weight (%)	0.50 ± 0.29	0.26 ± 0.13	0.38 ± 0.20
Relative liver weight (%)	5.52 ± 0.29	4.86 ± 0.17*	5.94 ± 0.44
Relative kidney weight (%)	1.82 ± 0.18	1.79 ± 0.15	1.78 ± 0.15
Relative heart weight (%)	0.47 ± 0.02	0.46 ± 0.05	0.45 ± 0.04
Relative brain weight (%)	1.07 ± 0.08	1.13 ± 0.04	1.08 ± 0.10
Relative lung weight (%)	0.52 ± 0.07	0.56 ± 0.04	0.63 ± 0.11
Relative stomach weight (%)	0.73 ± 0.07	0.69 ± 0.26	0.84 ± 0.15

*Significant difference compared with the control (*P* < 0.05). Values are mean ± SEM of 10 animals.

**Table 2:** Hematological profiles of mice following gavage administration of *C. baccatum* extracts for 60 days (200 mg/kg).

Parameters	Control	Ethanol	Butanol
Hb (g/dL)	13.1 ± 0.56	13.1 ± 0.95	13.1 ± 0.81
RBC count (10^6^/*μ*L)	8.5 ± 0.33	8.5 ± 0.43	8.3 ± 0.65
HCT (%)	40.1 ± 2.29	39.7 ± 3.53	39.1 ± 2.47
MCV (fl)	47.3 ± 1.57	46.6 ± 1.93	47.2 ± 1.82
MCH (pg)	15.5 ± 0.51	15.5 ± 0.44	15.8 ± 0.46
MCHC (g/dL)	32.9 ± 1.32	33.2 ± 0.72	33.5 ± 0.60
WBC count (10^3^/*μ*L)	6910 ± 2222.2	7818 ± 1639.2	8158 ± 2090.7
Lymphocyte (%)	74.5 ± 5.11	79.9 ± 7.06	70.3 ± 9.88
Neutrophils (%)	19.7 ± 7.0	12.2 ± 6.90	22.0 ± 7.97

Hb: hemoglobin; RBC: red blood cells; HCT: hematocrit; MCV: mean corpuscular volume; MCH: mean corpuscular hemoglobin; MCHC: Mean corpuscular hemoglobin concentration; WBC: white blood cells. No significant difference was observed among groups (*P* > 0.05). Values are mean ± SEM of 10 animals.

**Table 3:** Serum biochemical evaluations of mice following gavage administration of *C. baccatum* extracts for 60 days (200 mg/kg).

Parameters	Control	Ethanol	Butanol
ALT (IU/L)	29.0 ± 10.94	45.8 ± 40.52	42.2 ± 18.79
ALP (IU/L)	45.7 ± 21.74	28.5 ± 15.93	41.3 ± 14.68
Proteins (mg/dL)	4.9 ± 0.58	4.9 ± 0.42	4.8 ± 0.38
Albumin (mg/dL)	2.0 ± 0.45	2.0 ± 0.21	1.6 ± 0.38
LDH (IU/L)	150.2 ± 37.2	202.2 ± 83.93	148.0 ± 22.43
Triglycerides (mg/dL)	91.0 ± 25.63	76.4 ± 21.97	102.2 ± 31.42
Cholesterol (mg/dL)	129.5 ± 9.93	101.0 ± 13.58	119.4 ± 26.81
HDL-cholesterol (mg/dL)	63.5 ± 8.68	53.3 ± 7.69	56.7 ± 10.08
LDL-cholesterol (mg/dL)	47.8 ± 11.28	32.4 ± 11.08	42.3 ± 24.55
Glucose (mg/dL)	96.2 ± 44.45	133.5 ± 47.53	108.6 ± 34.70
Urea (mg/dL)	42.5 ± 3.15	45.6 ± 1.52	45.3 ± 12.53
Creatinine (mg/dL)	<0.20	0.20 ± 0.04	<0.20

ALT: alanine aminotransferase; ALP: alkaline phosphatase; LDH: lactate dehydrogenase. Neither of the treated groups was significantly different from the control group. Values are means ± SEM of 10 animals. No significant difference was observed among groups (*P* > 0.05).
